# Harveian Society of London

**Published:** 1889-04

**Authors:** 


### HARVEIAN SOCIETY OF LONDON.

*Meeting Held March 14th, 1889.*

THE PRESIDENT, THOMAS BUZZARD, M.D., F.R.C.P., IN THE CHAIR.

### ON PRE-CANCEROUS CONDITIONS OF THE TONGUE.

MR. BUTLIN in his paper dealt chiefly with three points: 1. The proportion of cases of cancer of the tongue in which the disease is preceded by a well-recognized pre-cancerous condition. 2. The relative importance of various pre-cancerous conditions. 3. The question of the early and free removal of some pre-cancerous conditions. In a certain number of cases which had been under the care of the author, cancer of the tongue had been preceded by a pre-cancerous condition in at least 70 per cent. Warty growths appeared to be the most dangerous of the conditions which actually and immediately precede cancer, and these warty growths were shown to be more frequent than is generally believed. The question was raised whether it would not be right, in cases of leucoma and chronic superficial glossitis, in which warts and warty growths form on the surface of the tongue, to remove the whole of the diseased area of the tongue, or certainly the fore part of the tongue, instead of merely removing the warty growth and an area of the surrounding tissue. Two cases were related in which simple warty growths formed on leukomatous tongues, and were removed, and in which at a later period cancer developed, but not in the seat of the removal of the warts. The use of liquor arsenicalis internally was recommended in all cases of chronic affection of the surface of the tongue, in which the disease is associated with various forms of chronic affection of the general integument (non-specific). Several cases were related to show the advantage of the removal of early cancerous affections of the tongue.

MR. JACOBSON said that he thought the pre-cancerous conditions were better marked in the tongue than in any other part, unless it might be occasionally in the breast. Yet sufficient use was not yet made of the knowledge of these pre-cancerous stages to remove the disease before the advent of the malignant stage. He believed it was most important for general practitioners to recognize these conditions that they might advise their patients to be operated upon in time. Mr. Jacobson showed a patient who had syphilitic leucoma of the tongue, with constant rawness and a small spot of induration. This was recognized as pre-cancerous and the half of the tongue removed. His speech now was but little affected. Mr. Jacobson strongly advocated the removal of one-half the tongue, and believed that the speech was always good after such an operation. Another patient was shown who had a pre-cancerous condition of the tongue, but declined operation for fear of interference with the practice of his profession as a player on a wind instrument. Mr. Jacobson had found it very difficult to persuade patients to be operated upon, unless they were convinced that the disease was actually cancerous. Mr. Jacobson also commented upon the pathological anatomy of these disorders. The affection was often latent for a time, but liable to sudden outbursts. Small operations gave rise to severe hæmorrhage which obscured the view of the operator and led to diseased portions being left behind.

Mr. Pickering Pick thought that in many cases chronic superficial glossitis in the various forms was a curable affection, and not every case required removal. He had found that the exhibition of arsenic in large doses was of remarkable use. At the same time the case must be watched carefully, and the disease removed freely on the slightest sign of epithelial change.

Mr. Barker agreed with Mr. Butlin in most points. He believed that leukoma once started was rarely recovered from. It might last a great number of years, but his experience was that it never changed except for the worst. For its relief he relied upon mild solvents, like carbonate of soda, which perhaps acted by rendering the secretions of the mouth less irritating, and promoted the exfoliation of epithelium. As long as the disease was pre-cancerous he would like to leave it alone, and remove it at the moment it became cancerous. He somewhat objected to the term pre-cancerous. As long as there was no induration, he thought the disease might be left alone, but the patient should be constantly watched by his medical adviser. Mr. Barker did not approve of removing wedge-shaped portions. It was better to remove the tongue within reasonable limits. Special attention should be paid in the operation to the deep dissection by the side of the tongue where the lymphatics ran. Epithelioma of the tongue had a great power of diffusing itself. Mr. Barker was not in favor of removing the tongue by halves, for the remainder of the tongue in such cases got bound down by scars.

Mr. Pye agreed with Mr. Barker as to partial removal. He asked whether it was not better to remove the whole tongue in cases of leukoma. However, he thought that the removed half of the tongue was not so bound down after partial removal as appeared to be the case. He also asked whether it were not possible to remove the superficial layer of the tongue when affected by leukoma.

Mr. Lockwood showed a specimen which exhibited leukoma and papilloma. Really pre-cancerous conditions might be partially removed, but true cancer should be removed as widely as possible.

Dr. Cleveland remarked on the conditions which predispose to cancer. Smokers were particularly liable, and he related several cases illustrating this, where early recognition and operation resulted in cure.

Dr. Drew asked whether ring-worm of the tongue was a pre-cancerous condition.

The President asked whether ulcers or clefts in patients who had suffered from syphilis were to be distinguished accurately as syphilitic, and successfully treated by iodide of potassium. He had seen such an ulcer heal for a time under the use of the drug, and afterwards recur as cancer. He remarked that epileptics were prone to bite the tongue constantly on one side, and he asked whether this had been noted as a precedent of cancer.

Mr. Butlin, in reply, said that there was undoubtedly a true syphilitic gummatous ulcer of the tongue which healed under iodide of potassium. He had seen a bite excite cancer. He thought that the majority of cancers ulcerated unless they were covered by horny epithelium. He had only seen one case of leukoma absolutely recover, and that under the use of arsenic. He thought that leukoma and superficial glossitis predisposed to cancer, but patients might have such conditions for many years without cancer developing. The wart on a leukomatous base never gets well, and always becomes cancerous. He thought it dangerous to attempt to cut away the superficial layer of the tongue in leukoma.—*The Medical Press*, April 3, 1889.

